# An Interactive Model of Target and Context for Aspect-Level Sentiment Classification

**DOI:** 10.1155/2019/3831809

**Published:** 2019-12-19

**Authors:** Hu Han, Guoli Liu, Jianwu Dang

**Affiliations:** ^1^School of Electronic & Information Engineering, Lanzhou Jiaotong University, Lanzhou 730070, China; ^2^Gansu Provincial Engineering Research Center for Artificial Intelligence and Graphic & Image Processing, Lanzhou 730070, China

## Abstract

Aspect-level sentiment classification aims to identify the sentiment polarity of a review expressed toward a target. In recent years, neural network-based methods have achieved success in aspect-level sentiment classification, and these methods fall into two types: the first takes the target information into account for context modelling, and the second models the context without considering the target information. It is concluded that the former is better than the latter. However, most of the target-related models just focus on the impact of the target on context modelling, while ignoring the role of context in target modelling. In this study, we introduce an interactive neural network model named LT-T-TR, which divided a review into three parts: the left context with target phrase, the target phrase, and the right context with target phrase. And the interaction between the left/right context and the target phrase is utilized by an attention mechanism to learn the representations of the left/right context and the target phrase separately. As a result, the most important words in the left/right context or in the target phrase are captured, and the results on laptop and restaurant datasets demonstrate that our model outperforms the state-of-the-art methods.

## 1. Introduction

The aspect-level sentiment classification is a fine-grained task in sentiment analysis [1]. Given a review and a target occurring in the review, it aims to identify the sentiment polarity (e.g., negative, neutral, or positive) expressed on each target in its context. For example, considering this review “*the voice quality of this phone is amazing*, *but the price is ridiculous*,” we observe that there are two targets (“*voice quality*” and “*price*”) with completely opposite polarities. The sentiment expressed on target “*voice quality*” is positive, whereas the sentiment for target “*price*” is negative. Jiang et al. [2] introduced a target-dependent Twitter sentiment classifier, which showed that not considering the target information discussed in the review results in 40% of sentiment classification errors. Therefore, the task of aspect-level sentiment classification is also aimed at predicting a sentiment category for a review-target pair.

Different from sentence- and document-level sentiment analysis [3–6], for aspect-level sentiment classification, a review may contain multiple review-target pairs, and thus, separating different contexts for different targets is a challenge. Many methods based on neural networks have been proposed for aspect-level sentiment classification. For example, Dong et al. [7] used the adaptive recursive neural network to evaluate the sentiments of specific targets in context words. Vo and Zhang [8] separated the whole review into three sections (the target, its left contexts, and its right contexts) and used neural pooling functions and sentiment lexicon to extract the feature vector for a given target. Tang et al. [9] divided the review into the left part with the target and the right part with the target and then used two long short-term memory (LSTM) networks to encode the two parts, respectively. Zhang et al. [10] used a gated neural network to capture the interaction information between the target and its surrounding contexts. To further focus on important words of the sentence that modulate the sentiment of the targets, Wang et al. [11] introduced LSTM networks and an attention mechanism to concatenate word representations with target embeddings to generate the final sentiment representations.

Although the previous approaches have realized the importance of targets in sentiment classification, these approaches only focus on the impact of targets on context modeling. How to use the interaction information between contexts and the target phrase to separately model contexts and targets has become a new research issue. Ma et al. [12] proposed an interactive attention network (IAN) that uses two LSTM networks to model the contexts and target phrase, respectively, and then uses the hidden states from the contexts to generate an attention vector for the target phrase, and vice versa. Based on [12], Huang et al. [13] proposed an attention-over-attention (AOA) neural network, which models targets and reviews simultaneously using two LSTMs and then the target representation and text representation can be interacted through the AOA module. Zheng and Xia [14] designed a left-center-right separated neural network to model the left context, target phrase, and right context, respectively, and modeled the relation between the target and the left/right context using a rotatory attention mechanism.

To further improve the representations of targets and contexts, we propose an interactive neural network model named LT-T-TR. Firstly, it divides a review into three parts: the left context with the target phrase, the target phrase, and the right context with the target phrase. Three Bidirectional Long Short-Term Memory networks (Bi-LSTMs) are used to model these parts, respectively. Secondly, different words in reviews have different contributions to the final representation, and contexts and targets are influenced by each other, so attention weights of the target phrase and the left/right context are computed by interactive attention between the target phrase and the left/right context. The process is made up of two parts: the first is target-to-context attention, which includes the target-to-left context attention and the target-to-right context attention, to get better representations of the left/right contexts; the second is context-to-target attention that includes the left context-to-target attention and the right context-to-target attention. After computing these attention weights, we get the target phrase and left/right context representations. Next, these representations are concatenated to generate the final classification vectors. Experimental results on laptop and restaurant datasets show that our method achieves obvious improvements. The main contributions of this study can be summarized as follows:

- Dividing a review into three parts: the left context with the target phrase, the target phrase, and the right context with the target phrase. Three Bi-LSTMs are used to model these parts, respectively.
- Computing attention weights of the left/right context and the target phrase and getting representations of the target phrase and the left/right context using attention weights.
- Concatenating these representations to form the final classification vectors and evaluating our model on laptop and restaurant datasets.

## 2. Model

**Figure 1:**
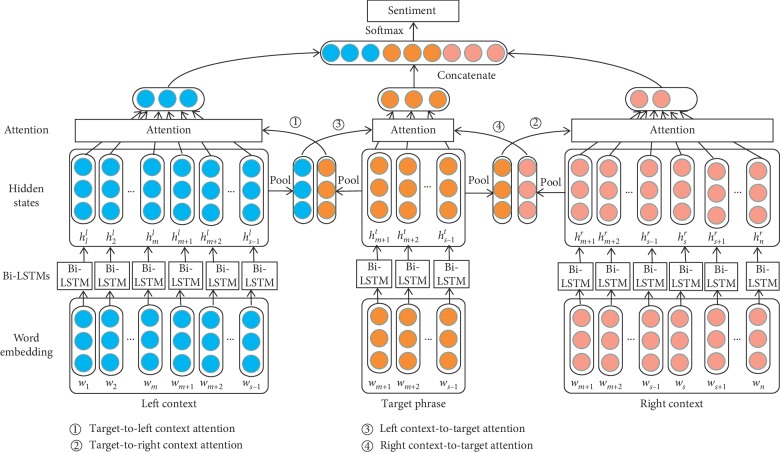
The overall architecture of our aspect-level sentiment classification model.

In this section, we first give the task definition of aspect-level sentiment classification. Afterward, we introduce the different components of our model as displayed in Figure 1.

### 2.1. Task Definition

Given a review *S*=[*w*_1_,…, *w*_*m*_, *w*_*m*+1_,…, *w*_*s*−1_, *w*_*s*_,…, *w*_*n*_] consisting of *n* words, *w*_1_, *w*_2_,…, *w*_*m*_ are the preceding context words, *w*_*m*+1_, *w*_*m*+2_,…, *w*_*s*−1_ are the target words, and *w*_*s*_, *w*_*s*+1_,…, *w*_*n*_ are the following context words. We divide the review into three parts: the left context LT=[*w*_1_^*l*^, *w*_2_^*l*^,…, *w*_*s*−1_^*l*^] consisting of *w*_1_, *w*_2_,…, *w*_*m*_ and *w*_*m*+1_, *w*_*m*+2_,…, *w*_*s*−1_, the target phrase *T*=[*w*_*m*+1_^*t*^, *w*_*m*+2_^*t*^,…, *w*_*s*−1_^*t*^] consisting of *w*_*m*+1_, *w*_*m*+2_,…, *w*_*s*−1_, and the right context RT=[*w*_*m*+1_^*r*^, *w*_*m*+2_^*r*^,…, *w*_*n*_^*r*^] consisting of *w*_*m*+1_, *w*_*m*+2_,…, *w*_*s*−1_ and *w*_*s*_, *w*_*s*+1_,…, *w*_*n*_. Aspect-level sentiment classification aims at determining the sentiment polarity of review *S* toward target *T*. For example, the sentiment polarity of review “*the voice quality of this phone is amazing*, *but the price is ridiculous*” toward target “*voice quality*” is positive, but the polarity toward target “*price*” is negative.

### 2.2. Bi-LSTMs

First, we represent each word in *S* as word embedding [15] and get word vectors [*v*_1_^*l*^, *v*_2_^*l*^,…, *v*_*s*−1_^*l*^] ∈ *R*^(*s* − 1)×*d*^, [*v*_*m*+1_^*t*^, *v*_*m*+2_^*t*^,…, *v*_*s*−1_^*t*^] ∈ *R*^(*s* − *m* − 1)×*d*^, and [*v*_*m*+1_^*r*^, *v*_*m*+2_^*r*^,…, *v*_*n*_^*r*^] ∈ *R*^(*n* − *m*)×*d*^ for LT, *T*, and RT, where *d* is the embedding dimension. Then, we feed these three-part word vectors to three Bi-LSTMs [16], respectively, to learn the hidden word semantics. Each Bi-LSTM is obtained by stacking a forward LSTM and a backward LSTM, which are good at learning long-term dependencies [17]. In the LSTM architecture, there are three gates (input gate, forget gate, and output gate) and a cell memory state. Each cell can be updated as follows:

(1) $$\begin{array}{c} X=\left[\begin{array}{c} h_{k-1}\\ v_{k} \end{array}\right],\\ i_{k}=\sigma \left(W_{i}\cdot X+b_{i}\right),\\ f_{k}=\sigma \left(W_{f}\cdot X+b_{f}\right),\\ o_{k}=\sigma \left(W_{o}\cdot X+b_{o}\right),\\ c_{k}=f_{k}⊙c_{k-1}+i_{k}⊙\;\tanh\left(W_{c}\cdot X+b_{c}\right),\\ h_{k}=o_{k}⊙\;\tanh\left(c_{k}\right), \end{array}$$

where *σ* is the sigmoid function, ⊙ denotes elementwise multiplication, and · stands for matrix multiplication; *W* and *b* denote the weight matrices and biases, respectively; *v*_*k*_ is the input word vector, and *h*_*k*−1_ is the previous hidden state.

For the left context LT, the input of Bi-LSTM is [*v*_1_^*l*^, *v*_2_^*l*^,…, *v*_*s*−1_^*l*^] ∈ *R*^(*s* − 1)×*d*^ and we get hidden states as follows:

(2) $$\begin{array}{c} \left[h_{1}^{l},h_{2}^{l},\ldots ,h_{s-1}^{l}\right]=\text{Bi}-\text{LSTM}\left(\left[v_{1}^{l},v_{2}^{l},\ldots ,v_{s-1}^{l}\right]\right), \end{array}$$

where the output *h*_*k*_^*l*^(*k*=1,…, *s* − 1) is obtained by concatenating the corresponding states of the forward and backward LSTM. Similarly, we can get the hidden semantic states [*h*_*m*+1_^*t*^, *h*_*m*+2_^*t*^,…, *h*_*s*−1_^*t*^] for target *T* and the hidden states [*h*_*m*+1_^*r*^, *h*_*m*+2_^*r*^,…, *h*_*n*_^*r*^] for the right context RT in the same way.

Then, through an average pooling operation, we can obtain the initial representations of LT, *T*, and RT as follows:

(3) $$\begin{array}{c} {\text{LT}}_{\text{initial}}=\overset{s-1}{\underset{k=1}{\sum }}\frac{h_{k}^{l}}{s-1}, \end{array}$$

(4) $$\begin{array}{c} T_{\text{initial}}=\overset{s-1}{\underset{k=m+1}{\sum }}\frac{h_{k}^{t}}{s-m-1}, \end{array}$$

(5) $$\begin{array}{c} {\text{TR}}_{\text{initial}}=\overset{n}{\underset{k=m+1}{\sum }}\frac{h_{k}^{r}}{n-m}. \end{array}$$

### 2.3. Attention Layer

After getting the hidden representations of the context and the target phrase generated by three Bi-LSTMs, we use the attention mechanism to calculate the different importance of words in the left/right context and the target phrase.

#### 2.3.1. Target-to-Context Attention

Given the hidden representations of the left context [*h*_1_^*l*^, *h*_2_^*l*^,…, *h*_*s*−1_^*l*^] and the average representation of target *T*_initial_, we first get the *target-to-left context attention* representation LT_final_ by

(6) $$\begin{array}{c} {\text{LT}}_{\text{final}}=\overset{s-1}{\underset{k=1}{\sum }}\alpha_{k}^{l}h_{k}^{l}, \end{array}$$

where *α*_*k*_^*l*^ is the weight of *h*_*k*_^*l*^ that we can obtain from a softmax function:

(7) $$\begin{array}{c} \alpha_{k}^{l}=\frac{\exp\left(f_{\text{att}}\left(h_{k}^{l},T_{\text{initial}}\right)\right)}{\sum_{j=1}^{s-1}\exp\left(f_{\text{att}}\left(h_{j}^{l},T_{\text{initial}}\right)\right)}. \end{array}$$

Here, *f*_att_ is a score function that indicates the importance of words in the left context influenced by the target:

(8) $$\begin{array}{c} f_{\text{att}}\left(h_{k}^{l},T_{\text{initial}}\right)=\tanh\left(h_{k}^{l}\cdot W_{a}\cdot T_{\text{initial}}^{T}+b_{a}\right), \end{array}$$

where *tanh* is a nonlinear function, *W*_*a*_ is the weight matrix, *b*_*a*_ is the bias, and *T*_initial_^*T*^ is the transpose of *T*_initial_.

Similar to equations (6)–(8), we can also obtain the target-to-right context attention representation TR_final_ using the average representation of the target *T*_initial_.

#### 2.3.2. Context-to-Target Attention

For the hidden representations of target [*h*_*m*+1_^*t*^, *h*_*m*+2_^*t*^,…, *h*_*s*−1_^*t*^], we first compute the weight representations as follows:

(9) $$\begin{array}{c} \alpha_{k}^{lt}=\text{soft}\max\left(f_{\text{att}}\left(h_{k}^{t},{\text{LT}}_{\text{initial}}\right)\right), \end{array}$$

(10) $$\begin{array}{c} f_{\text{att}}\left(h_{k}^{t},{\text{LT}}_{\text{initial}}\right)=\tanh\left(h_{k}^{t}\cdot W_{L}\cdot {\text{LT}}_{\text{initial}}^{T}+b_{L}\right), \end{array}$$

where *W*_*L*_ and *b*_*L*_ are the weight matrix and bias, respectively.

Then, through calculating the weighted combination of the hidden states of the target phrase, we can obtain the left context-to-target representation as follows:

(11) $$\begin{array}{c} T_{\text{final}}^{\text{lt}}=\overset{s-1}{\underset{k=m+1}{\sum }}\alpha_{k}^{lt}h_{k}^{t}. \end{array}$$

Similar to equations (9)–(11), we can obtain the right context-to-target representation *T*_final_^rt^ by using TR_initial_ and the hidden representations of the target.

After getting *T*_final_^lt^ and *T*_final_^rt^, we get the final representation of the target phrase through concatenating *T*_final_^lt^ and *T*_final_^rt^:

(12) $$\begin{array}{c} T_{\text{final}}=\left[T_{\text{final}}^{\text{lt}};T_{\text{final}}^{\text{rt}}\right]. \end{array}$$

### 2.4. Final Classification

Then, we concatenate LT_final_, *T*_final_, and TR_final_ as the final representation of review *S*:

(13) $$\begin{array}{c} v=\left[{\text{LT}}_{\text{final}};T_{\text{final}};{\text{TR}}_{\text{final}}\right]. \end{array}$$

We project *v* into the space of targeted *C* classes through a nonlinear function:

(14) $$\begin{array}{c} x=\tanh\left(W_{v}\cdot v+b_{v}\right), \end{array}$$

where *W*_*v*_ and *b*_*v*_ are the parameters. Finally, the sentiment polarity of the review *S* with sentiment polarity *c* ∈ *C* toward a target *T* is calculated as follows:

(15) $$\begin{array}{c} P\left(y=c\right)=\frac{\exp\left(x_{c}\right)}{\sum_{i\in C}\exp\left(x_{i}\right)}. \end{array}$$

### 2.5. Model Training

The model is trained in an end-to-end way. The loss function is the crossentropy error:

(16) $$\begin{array}{c} \text{loss}=-\underset{\left(S,T\right)\in D}{\sum }\underset{c\in C}{\sum }g\left(y_{\left(S,T\right)}=c\right)\cdot \;\log\left(P\left(y_{\left(S,T\right)}=c\right)\right), \end{array}$$

where *D* means all training data, (*S*, *T*) means a review-target pair, *C* is the number of categories of sentiment, *P*(*y*_(*S*, *T*)_=*c*) is the probability of predicting (*S*, *T*) as class *c* given by the softmax function, and *g*(*y*_(*S*, *T*)_=*c*) shows whether class *c* is the correct sentiment category.

## 3. Experiment

### 3.1. Experimental Settings

#### 3.1.1. Datasets

We conduct our experiments using the dataset for SemEval 2014 Task 4 [18]. This dataset contains customer reviews on restaurants and laptops. Each review has one or more targets with their corresponding polarities. The polarity of targets can be positive, negative, neutral, or conflict. However, we only consider the first three labels for classification. The statistics of the datasets are shown in Table 1.

#### 3.1.2. Parameters and Evaluation Metric

In our experiments, the dimensions of word embeddings, attention vectors, and LSTM hidden states are set to 300. All word embeddings are initialized by GloVe [19], and we randomly initialize the out-of-vocabulary words from uniform distribution *U*(−0.1, 0.1). All weight matrices are randomly initialized from uniform distribution *U*(−0.1, 0.1), and all bias terms are set to zero. The dropout rate is set to 0.5.

We adopt the accuracy to evaluate the performance of our model, which is defined as follows:

(17) $$\begin{array}{c} \text{Acc}=\frac{T}{N}, \end{array}$$

where *T* is the number of correctly predicted samples and *N* is the total number of samples.

### 3.2. Model Comparisons

We compare our model with some baseline approaches:

  Majority: the largest sentiment polarity in the training set is regarded as the classification result of each sample in the test set.
  LSTM: a standard LSTM which models the review as a whole and uses the last hidden state of LSTM as the final revive representation [9].
  TD-LSTM: TD-LSTM obtains the final sentiment representation by concatenating two LSTM networks which model the preceding and following contexts surrounding the target, respectively [9].
  AE-LSTM: AE-LSTM concatenated the target vector with each word in review as the input of LSTM [11].
  ATAE-LSTM: ATAE-LSTM appends the aspect embedding into each word vector to strengthen the importance of the target [11].
  IAN: two LSTM networks are used to model the review and target phrase, respectively. It uses the hidden states of the review to generate an attention vector for the aspect, and vice versa. Based on these two attention vectors, it outputs a review representation and an aspect representation for classification [12].

The experimental results are shown in Table 2.

First, the worst method is Majority, demonstrating that for aspect-level sentiment classification, a powerful feature representation is important. Then, among all the other methods based on LSTM, the basic LSTM approach has the worst performance because it just models the whole review and ignores the target information. TD-LSTM has an improvement of 1% on the restaurant dataset and 2% on the laptop dataset over LSTM when target information is taken into consideration. Because the attention mechanism is introduced, AE-LSTM and ATAE-LSTM perform better than TD-LSTM. IAN obtains better results on restaurant and laptop datasets than LSTM-based methods because IAN explores separate representations of targets and interactive learning between the context and target. Our LT-T-TR model significantly surpasses the performance of IAN and all other baseline approaches. This reinforces our hypothesis that a model capable of capturing target-context dependencies interactively indeed performs better. We will conduct a more detailed analysis in the following sections.

### 3.3. Model Analysis: The Effect of Different Pooling Functions

In this section, we analyze the contribution of various pooling functions (see equations (3)–(5)) by using the LT-T-TR model. The results are shown in Table 3. It can be seen that the accuracy (77.5%) is the lowest when using min pooling alone to extract hidden features. By using max and avg pooling, the model has a significantly improved accuracy (79.3% and 79.6%, respectively). Finally, we obtain the best accuracy (80.6%) by combining max and avg pooling.

### 3.4. Model Analysis: The Effect of Different Sequence Models

We analyze the effect of different sequence models, recurrent neural networks (RNN), LSTM, and gated recurrent unit (GRU), to verify the effectiveness of our model. The results of experimental comparison results are shown in Table 4. We can see that LSTM performs better than RNN, and this is because LSTM has more complicated hidden units and offer better computation capability than RNN. Simultaneously, GRU has fewer parameters to train compared to LSTM, so that GRU has better accuracy than LSTM. Bi-LSTM has slightly better performance compared to GRU and LSTM because Bi-LSTM can capture more context semantic information than LSTM and GRU.

### 3.5. Model Analysis

To validate the effectiveness of the LT-T-TR model, we design several models in this section. We first input the review as a whole (rather than as three segments) into Bi-LSTM for modeling, and then use the attention mechanism to calculate the importance of each word toward sentiment categories. We refer to this model as No-Separation. Second, we simplify the LT-T-TR model by using the average of initial target vectors to represent the target phrase, we refer to this model as No-Target-Learned.

Furthermore, we compare the effect of interactive attention modeling between the target and left/right context. First, we build a model (named No-Interaction) without interactive information by removing the attention interaction operation between the left/right context and target phrase and just learn the attention weight representation by their own Bi-LSTM hidden states. Then, we build the Target-to-Context model by removing context-to-target attention, which is based on Target-to-Context [12]. Finally, we create an L-T-R model through dividing a review into the preceding context (without target), the target, and the following context (without target) and then model these three parts in the same way as in the LT-T-TR model.

Table 5 shows the experimental results. It can see that the No-Separation model achieves the worst performance among all approaches, and the No-Target-Learned model performs worse than No-Interaction and Target-to-Context model. This verifies that the target representation is important to judge the final sentiment categories, and the target should be modeled separately.

And L-T-R and LT-T-TR perform better than the No-Interaction model and the Target-to-Context model, which shows that the interaction between the target phrase and left/right context is important to final sentiment classification. Moreover, L-T-R has slightly worse results than the LT-T-TR model because the target phrase is not contained in the left/right context.

### 3.6. Qualitative Analysis

**Figure 2:**
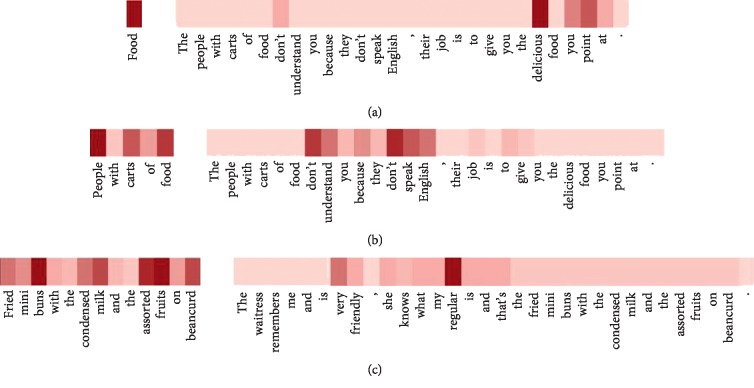
Attention visualizations.

In this section, we selected three examples from the restaurants dataset to analyze which words contribute the most to the final classification. We get the attention weights and then visualize them by using a visualization tool Heml [11]. The results are shown in Figure 2, in which the color depth represents the importance of a word: the darker, the more important.

The review in Figures 2(a) and 2(b) is “*The people with carts of food don't understand you because they don't speak English*, *their job is to give you the delicious food you point at*.” The corresponding targets are “*food*” and “*people with carts of food*,” respectively. It can be seen that when a review contains two targets, the correct sentiment categories for each target can be calculated automatically through our model, that is, the attention mechanism can dynamically obtain the important words from the whole review. In Figure 2(b), we can see that “*people*” is the most important word in the target phrase “*people with carts of food*.” In Figure 2(c), the target is a multiword phrase “*fried mini buns with the condensed milk and the assorted fruits on beancurd*” and “*buns*” and “*fruits*” are more important than other words, so our model pays more attention to “*buns*” and “*fruits*.” This also proves that just averaging the vectors of words the target phrase contains to represent the target does not help much. Therefore, modeling the target phrase and context interactively is important for aspect-level sentiment classification.

### 3.7. Error Analysis

We made an error analysis of the experimental results. The first type of error is caused by noncompositional sentiment expression [20]. For instance, in this review “*not only was the look of the food fabulous*, *but also the taste was to die for*,” “*taste*” is a target and “*to die for*” is the relevant sentiment expression, whose meaning should not be understood literally. The second kind of error comes from complex sentimental relation expressions such as double negatives, assumptions, and comparisons, like “*even though the price of this camera is unacceptable*, *I love its lens*.” Our model fails to deal with the complex sentiment expression in this case. Furthermore, in the review “*the movie was really on point—I was surprised*,” “*movie*” is the target word and the idiom “*on point*” is the relevant sentiment expression, which is difficult to be identified by our model.

## 4. Related Work

### 4.1. Aspect-Level Sentiment Classification

Sentiment analysis, also known as opinion mining [1, 21], has brought the widespread attention from both industry and academic communities. As a fine-grained task in the field of sentiment analysis [1], aspect-level sentiment classification has drawn a lot of attention, which is also considered as a kind of text classification problem. Traditional text classification methods depend greatly on the effectiveness of the feature engineering [22], which lacks generalization and is difficult for us to discover the potential explanatory or discriminative factors of data. In recent years, distributed word representation and neural network methods have been proposed and shown promising performance on this task [7, 8]. Dong et al. [7] used an adaptive recursive neural network to evaluate the sentiments of specific targets in context words. Vo and Zhang [8] separated the whole review into three sections (the target, its left contexts, and its right contexts) and used neural pooling functions and sentiment lexicon to extract the feature vector for a given target.

### 4.2. Neural Network for Aspect-Level Sentiment Classification

Today, neural network approaches are extremely fashionable for many natural language processing tasks and obviously, the field of sentiment classification is no exception. Many sentence/document-level sentiment classification tasks are dominated by neural network architectures [23–25]. To further incorporate context information with target information, several models have been proposed, such as the target-dependent LSTM [9], which models each sentence toward the aspect. ATAE-LSTM and AT-LSTM [11] are attentional models inspired by [26]. AT-LSTM can be considered as a modification of the neural attention proposed in [26] for entailment detection, swapping the premise's last hidden state for the aspect embedding. Han et al. [27] proposed a novel neural network based on LSTM and the attention mechanism for word context extraction and document representation. Chen et al. [28] combined regional long short-term memory and convolutional neural network for target-based sentiment classification. Zhang et al. [29] introduced dynamic memory networks based on multiple attention mechanism and LSTM, which showed a significant performance in aspect-level sentiment classification. Yang et al. [30] designed a coattention-LSTM network based on coattention mechanism for aspect-based sentiment analysis, combining the target and context attention vectors of sentences. The work most relevant to ours is IAN [12], which models the sentence and aspect term using two LSTM networks, respectively. It uses the hidden states from the sentence to generate an attention vector for the aspect, and vice versa. Based on these two attention vectors, it outputs a sentence representation and an aspect representation for classification.

Despite these aforementioned methods are effective, discriminating different sentiment polarities for different targets is still a challenging issue. Therefore, it is necessary to design a powerful neural network for aspect-level sentiment classification.

## 5. Conclusions

In this study, we have proposed an interactive neural network for aspect-level sentiment classification. The approach uses Bi-LSTM and an attention mechanism to interactively learn the important words in the target and context and generates the review representation for the final sentiment classification. Experimental results on the SemEval 2014 dataset show that our method achieves significant improvements. Our model analysis also shows that different sequence models can discriminatively learn the important words in the context and in the target. Furthermore, our model cannot handle several error cases effectively.

## Figures and Tables

**Table 1:** The statistics of the datasets.

Dataset	Positive	Neutral	Negative
Laptop-train	994	464	870
Laptop-test	341	169	128
Restaurant-train	2164	637	807
Restaurant-test	728	196	196

**Table 2:** Comparison results. Accuracies for three-way classification on the restaurant and laptop datasets.

Method	Restaurant	Laptop
Majority	0.535	0.650
LSTM	0.743	0.665
TD-LSTM	0.756	0.681
AE-LSTM	0.762	0.689
ATAE-LSTM	0.772	0.687
IAN	0.786	0.721
LT-T-TR	0.806	0.743

**Table 3:** The effect of different pooling functions.

Pooling function	Restaurant
Min	0.775
Max	0.793
Avg	0.796
Max + avg	0.806

**Table 4:** Comparison results.

Method	Restaurant	Laptop
LT-T-TR (RNN)	0.776	0.690
LT-T-TR (LSTM)	0.788	0.726
LT-T-TR (GRU)	0.790	0.730
LT-T-TR (Bi-LSTM)	0.806	0.743

**Table 5:** Analysis of our LT-T-TR model.

Method	Restaurant	Laptop
No-Separation	0.758	0.684
No-Target-Learned	0.760	0.707
No-Interaction	0.776	0.713
Target-to-Context	0.785	0.722
L-T-R	0.795	0.730
LT-T-TR	0.806	0.743

## Data Availability

The data used to support the findings of this study are included within the article.
